# Self-Procured Abortion

**Published:** 1909-07-17

**Authors:** 


### Self-procured Abortion.

Two weeks ago attention was called in these columns to the interruption for ten years of the career of a man who devotes a considerable part of his energies to the procuring of abortion by illegal operations. Satisfactory as the facts of his conviction and sentence are, it may be questioned whether the evil wrought by those who carry on these practices is anything like so great as that resulting from the indiscriminate drug-swallowing in which so many women who are, or suspect themselves to be, pregnant indulge with the object of aborting. Such at least is the inference suggested by some most remarkable evidence given at an inquest in Grimsby on July 7. Briefly, a fisherman's wife in that town died after abortion in circumstances suggesting that the cause of death was poisoning by lead, a suspicion confirmed by autopsy. It was proved in evidence that the deceased, who had had three children, had informed her mother that she had decided to terminate her fourth pregnancy by taking drugs, and that the mother had unsuccessfully endeavoured to dissuade her from this course. It is therefore established beyond question that the woman died as the result of the self-administration of lead taken to procure abortion. The doctor who was called in to the case stated in Court that this practice is very common in Grimsby, and is not confined to the working classes; he has himself been called in to as many as five cases within twenty-four hours in which this drug has been used and has brought about the desired effect. It is difficult to conceive the condition of affairs in which one practitioner can be sent for to five cases of procured abortion in one day; even granting that coincidence may have had something to do with so formidable a total, yet the use of lead compounds for the termination of pregnancy must be rife indeed among the population when such an amazing day's work can confront one man.

In making this experience public the witness alluded in emphatic tones to the fact that the use of lead in doses sufficient to cause abortion is attended by very considerable risks to the mother of insanity, paralysis, anæmia, and kidney diseases; and this point was driven home by the Coroner in his summing up to the jury. It has, unfortunately, become more or less public property that lead compounds in sufficient doses are very reliable abortifacients. What is much less well known to the public is that a dose sufficient to poison the *fœtus in utero* is sufficient also to poison the mother; and that, though it is only in exceptional cases that her death immediately results, yet the other consequences of this method of terminating pregnancy are by no means trivial, but often very serious. Disquieting as the revelations of the medical witness are, it is still more significant that the Coroner stated that many other practitioners in Grimsby have similar experiences to record. That occasional attempts at abortion or suicide should be made by the half-distraught victims of seduction is to be expected as long as human nature remains what it is; but that wholesale self-drugging by an enormous proportion of expecting mothers should be possible without any effort at prevention is both a reproach to and a very serious matter for the State. The moral and economic sides of this question are large and difficult, and cannot now be discussed; but at least it is matter for congratulation that the Grimsby jury did its duty by returning a verdict of *felo de se*. How far this will act as a deterrent to others is a subject for mere conjecture; but it is to be hoped that any good results that follow will not be confined to Grimsby.

